# The Incidence of Inflammatory Bowel Disease in the Paediatric Population in the District of Lower Silesia, Poland

**DOI:** 10.3390/jcm10173994

**Published:** 2021-09-03

**Authors:** Elzbieta Krzesiek, Anna Kofla-Dlubacz, Katarzyna Akutko, Andrzej Stawarski

**Affiliations:** 2nd Department and Clinic of Paediatrics, Gastroenterology and Nutrition, Medical University of Wroclaw, M.Curie-Sklodowskiej St. 50/52, 50-369 Wroclaw, Poland; anna.kofla-dlubacz@umed.wroc.pl (A.K.-D.); katarzyna.akutko@umed.wroc.pl (K.A.); andrzej.stawarski@umed.wroc.pl (A.S.)

**Keywords:** inflammatory bowel disease, incidence, Poland

## Abstract

The incidence of IBD has been rising over the last decades. The trend applies not only to well-developed countries but also to the regions with limited number of cases so far, e.g., Asia or Middle East. Aim: The aim of the study was to determine the incidence of paediatric IBD in the district of Lower Silesia, Poland, between 2016 and 2018. Methods: The number of newly diagnosed IBD per 100,000 children, living in the region, was calculated. The characteristics of the group were established. Results: There were 81 cases of paediatric IBD diagnosed between the 1st of January 2016 and 31st of December 2018. The diagnosis of ulcerative colitis (UC) was made for 42 children. In the same period of time 39 cases of Crohn disease (CD) were recognised. The incidences were calculated as 2.57 for UC, and 2.38 for CD. The total incidence of IBD between 2016 and 2018 was 4.96/100,000/year which is rise in the last 20 years. Conclusion: An increase in incidence of IBD in the district of Lower Silesia has been observed.

## 1. Introduction

Inflammatory bowel diseases (IBDs) are chronic, progressive conditions of the digestive tract, with periods of exacerbations and remission. These include ulcerative colitis (UC), Crohn’s disease (CD), IBD-unclassified (IBD-U)**,** and other rare conditions, e.g., microscopic colitis, eosinophilic colitis, or Behcet disease. The aetiology of IBD is multifactorial, and the involvement of genetic, immunological, and environmental factors has been proven to play a crucial role in the initiation and maintenance of the disease. The highest incidence of IBD is noted in young adults—in the third and fourth decade of life, in the case of CD, and the fourth and fifth decade, in the case of UC. About 20 to 25% of cases are related to the paediatric population; among them, 15% is the early onset IBD for children under the age of 5 years.

Among adults, men are more likely to develop UC, when in the group of male adolescents CD is more frequently diagnosed [1,2,3].

From the 1950s, in most geographic regions, the number of patients with newly recognised IBD has significantly risen in all age groups. The trend applies not only to well-developed countries, traditionally matched with a high incidence of IBD. The increase has also been observed in Asia and the Middle East [1,4,5,6].

The highest incidence of UC has been currently recorded in Europe (24.3/100,000), while the new cases of CD have been mainly recognised in North America (20.2/100,000). Nevertheless, Europe still remains the region with the highest prevalence of both UC and CD that ranges, respectively, 505/100,000 for UC and 322/100,000 for CD. However, North America is located in second place, with 248.6 cases for 100,000 of UC and 318.5 per 100,000 of CD. The incidence and prevalence of IBD are much lower in Asia and the Middle East (incidence: UC 6.3/100,000, CD 5/100,000; prevalence: UC 168.3/100,000 and CD 67.9/100,000), although dynamic growth has been noted in the previous years.

In Western Europe, a significant increase in the incidence of UC was observed in the second half of the 20th century, when it reached a plateau. In Eastern Europe, the uptrend is still present, the same as in Asia and Africa. The rise in the incidence of UC has preceded the increase in CD for about 10 years, and the rise of newly diagnosed cases of CD has been recently observed in Canada and New Zealand. Data on incidence in Poland still place our country among those with a low incidence of IBD (in 2007 incidence of 12.8/100,000 and CD 4.2/100,000).

This only applies to the adult population. The incidence of CD in children is growing and reaches 60% of all IBD cases in Europe and North America. In Poland, the recognition of UC still dominates among IBD patients. (1.3 vs. 0.6/100,000) [1,4,5,6,7,8,9].

The aetiology of IBD is not fully understood. Immunological, genetic, and environmental factors play important roles in the initiation and maintenance of the disease. The change in dietary habits, lifestyle, food additives, as well as environmental pollution, may all have an impact on the increase of IBD cases in developing countries. Varied geographical distribution and differences between ethnic groups confirm an involvement of genetic factors in IBD. Family occurrence is estimated at 2 to 14%, in CD cases, and 8 to 14%, in patients with UC. [1,4,10,11]. In 2001, the mutation NOD2/CARD15 on the 16th chromosome was proven to be involved in the higher risk of developing CD. Mutations in over 163 loci increasing the risk of CD have been described previously, but still, genetic factors seem to be more crucial in early onset IBD, while in older children, the aetiology is more multifactorial. Nevertheless, access to diagnostic tools and awareness among doctors play essential roles in the higher recognition of IBD in the general population [2,3,11,12,13,14].

## 2. Aim

The aim of this work was the estimation of the incidence and prevalence of IBD (UC and CD) in the region of Lower Silesia, Poland (south Poland with a population of 3 million citizens). The impact of age, place of living, as well as the analysis of main symptoms in both UC and CD, was taken into consideration.

## 3. Materials and Methods

In the study, the incidence (morbidity) of UC and CD in the region of Lower Silesia, Poland, between 1 January 2016 and 31 December 2018 was determined.

Diagnosis of IBD was based on clinical symptoms, laboratory parameters, endoscopic/histologic features, and results of imaging tests (magnetic resonance enterography), with the use of the Porto criteria [15]. The number of newly registered cases of these diseases during the year per 100,000 people in the paediatric population (between 2 and 18 years of age) was calculated. For this purpose, the number of inhabitants available on the website of the Central Statistical Office [16] was used.

In the group of children with IBD, the age at the time of diagnosis, the gender, the period from first symptoms to the diagnosis, as well as the place of residence (village, city), the pattern of symptoms, and seasonality of the disease were estimated.

## 4. Statistical Analysis

The results of the research were statistically analysed (Statistica 13PL program, StatSoft, Poland Inc.). Descriptive statistics measures were calculated: means and median values, both accompanied by 95% confidence interval (95% CI), standard deviation (SD), and ranges of minima and maxima. Statistical significance of differences between the nominal features was calculated with the Pearson χ^2^ test, with the Yates χ^2^ correction in case of insufficient values of the expected multiplicities in individual subgroups. To compare the two independent variables Mann–Whitney U test was used. *p* values of <0.05 were considered significant.

## 5. Results

From 1 January 2016 to 31 December 2018 in the clinic, 92 children were diagnosed with IBD, and 17 children were excluded from the study because they lived outside Lower Silesia. During this period, an additional six patients, residents of Lower Silesia, were referred to the clinic immediately after diagnosis in regional hospitals. Finally, 81 patients were analysed: 40 girls and 41 boys, only residents of the Lower Silesia Province. The youngest patient at the time of diagnosis was 2 years and 6 months old, and the oldest was 17 years and 7 months old.

Within 3 years, the diagnosis of UC was made in 42 children (51.9%), aged 3.6 to 17.7 years, average age 13.2 years, SD 3.6 (25 girls, mean age 13.6 years, SD 3.2; 17 boys, mean age 12.5 years, SD 4.2). Time to diagnosis from the first symptoms of the disease ranged from 1 week to 17 months, mean length 3.7 months, and SD 3.2.

The diagnosis of CD was made in 39 children (48.1%), aged 2.6 to 17, mean age 13.1 years, SD 3.9 (15 girls, mean age 13.9 years, SD 2.7; 24 boys, mean age 12.5 years, SD 4.6), and the time to diagnosis ranged from 1 to 60 months, mean length 6.9 months, and SD 9.5. The peak of the incidence in adolescent age (10–17 years of age) was observed and the tendency applied to both UC (73.8%) and CD (69.2%) (Table 1).

Clinical and endoscopic activity, their body mass index (BMI), as well as disease localisation in IBD patients, are presented in Table 2 and Table 3.

The incidence, i.e., the number of newly registered cases of the disease during the year, per 100,000 people living in the region of Lower Silesia aged 2 to 18 years old was 2.57 for UC and 2.38 for CD (Table 4).

Girls and boys were represented as IBD patients equally in the studied population (40 girls—49.4% and 41 boys—50.6%). Girls more frequently tended to develop UC (59.5%), while boys were more often diagnosed as CD (61.6%); however, the difference was not statistically important (*p* = 0.058).

**Figure 1:**
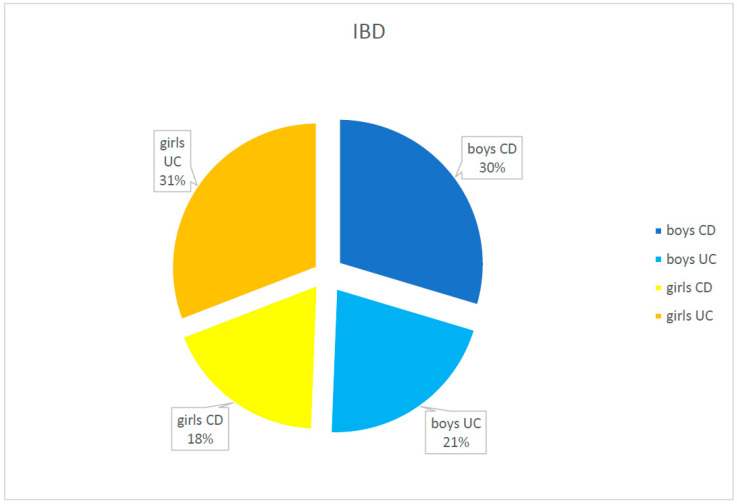
Gender structure: UC—ulcerative colitis; CD—Crohn’s disease; IBD—inflammatory bowel disease.

**Figure 2:**
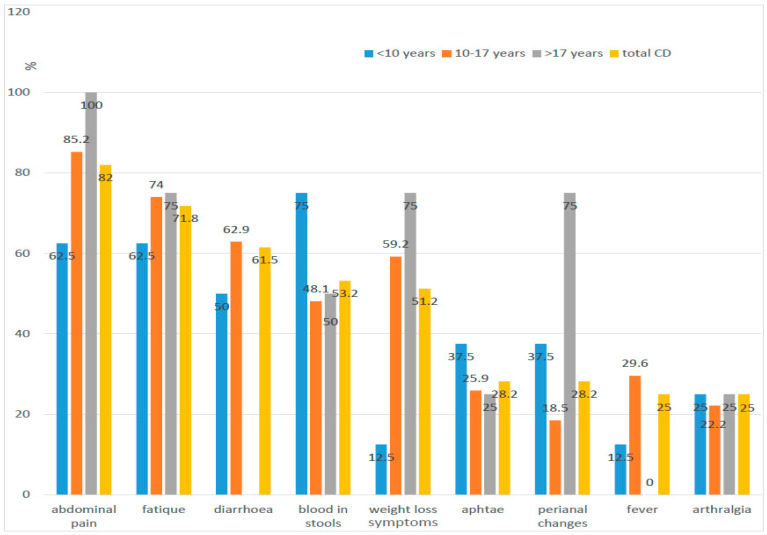
Correlation between symptoms and age: CD—Crohn’s disease.

The most common symptoms of UC were blood in the stool (95.2%), diarrhoea (80.9%), and abdominal pain (76.2%). In the case of CD, these were abdominal pain (82%), weakness (71.8%, and diarrhoea (61.5%) (Figure 1 and Figure 2).

In the study, no statistically significant difference was found in the incidence of diarrhoea and abdominal pain (*p* = 0.0528; *p* = 0.5175) and parenteral manifestation within the joints and skin involvement (*p* = 0.469; *p* = 0.0532) between patients with UC and CD. The presence of blood in the stool appeared to be the only symptom that occurred more frequently in patients with UC than in patients with CD (*p* = 0.0002). On the other hand, weakness, weight loss, changes in the oral cavity and in the upper gastrointestinal tract, as well as perianal changes, were more common in patients with CD than UC, and the observed differences were statistically significant (Table 5).

**Figure 3:**
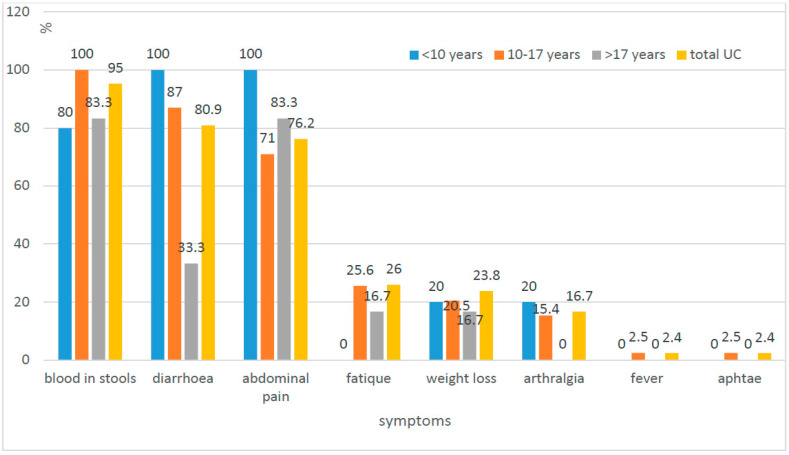
Correlation between symptoms and age (UC—ulcerative colitis).

The first symptoms of IBD occurred mostly in the winter period, from December to January, and in early summer, from May to June (Figure 3).

**Figure 4:**
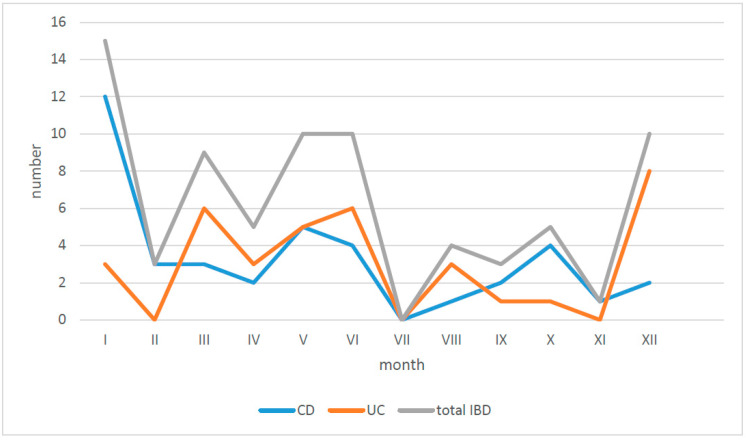
Seasonality of inflammatory bowel disease in children: UC—ulcerative colitis; CD—Crohn’s disease; IBD—inflammatory bowel disease.

In the studied group, 35.7% of patients with UC were residents of small towns (less than 20 thousand inhabitants), 38.5% of CD patients were living in cities up to 20 thousand people (20–100,000) (Figure 4). There is no significant difference in the incidence of CD/UC taking into account the place of living (*p* > 0.05) (Table 6).

**Figure 5:**
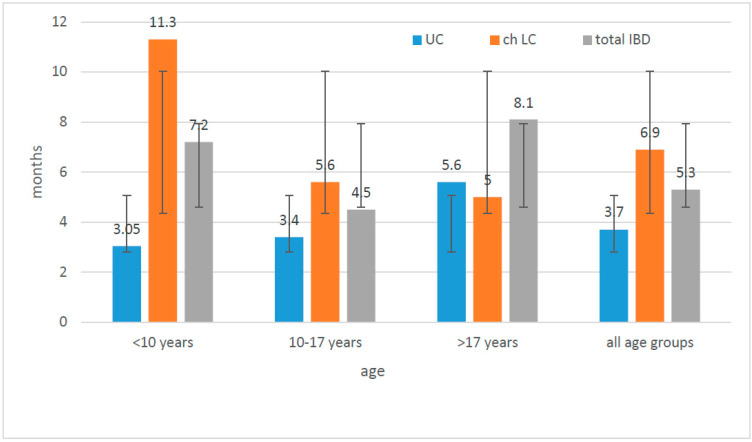
Correlation between time to diagnosis and patient’s age: *n*-number of children; *n* < 10 years: UC-5; CD-8; *n =* 10—17 years: UC-31; CD-27; *n >* 10 years: UC-6; CD-4; UC—ulcerative colitis; CD—Crohn’s disease; IBD—inflammatory bowel disease.

The time from the onset of symptoms to diagnosis was longer in all age groups (except patients >17 years of age) for CD than for UC (the difference was statistically significant, U-Mann–Whitney test, *p* = 0.008). The diagnosis was settled earlier in the case of patients from villages and small cities than in other subgroups. Children from cities larger than 20,000, but less than 100,000 went through the longest diagnostic process to the final diagnosis. (Figure 5).

## 6. Discussion

The analysis of the incidence of IBD in children living in the region of Lower Silesia confirms the global trend of increasing incidence of IBD, especially CD. In the years 1998–2000, the first study of the incidence among the paediatric population of Lower Silesia was performed. Comparing the obtained results with the data from the previous one, a rise in the incidence of IBD from 61/36 months to 81/36 months was observed. The increase from 61 to 81 cases within 36 months was shown, with an over fourfold growth in the case of CD, from 9 to 39 cases within 3 years [17] (statistically important, *p* < 0.05). In the analysed population, the number of cases of UC was slightly higher—51.9% compared to CD (48.1%) (no statistical difference, *p* > 0.05). The frequency in both genders was equal (50.6% boys vs. 49.4% girls, *p* > 0.05). Although boys were more likely to develop CD (61.5%) and girls UC (59.5%), no statistically important differences were detected (*p* = 0.058).

In the previous 3-year follow-up of children with IBD (1998–2000), there were 48.9% of boys and 51.1%, girls. The boys in 66.7% of the cases were diagnosed as CD and the girls in 52.2% of the cases as UC [17].

Family history (first-degree relatives with IBD) was positive for 7.1% of children with UC. Overall, 20% of those patients were diagnosed before the age of 10 which seems to confirm the role of genetic factors in the aetiology of early onset IBD. However, we could not observe this kind of in cases of CD; family history was positive for only 5.1% of children. Moreover, all of them were diagnosed between the ages of 10 and 17. Further observation is needed as the study groups were limited (Table 1).

There were statistically significant differences in the frequency of clinical symptoms presented by patients with UC and CD. Blood in the stools was observed in over 95% of patients with UC and only in half of the patients with CD (53%). The exclusion of the group of children with CD recognised under the age of 10 was made. The blood in stools as the first symptom occurred in this group more frequently despite the final diagnosis of CD (62.5% under the age of 10 to 48.4% over the age of 10, *p* < 0.05), which matches the typical lower location of changes in small children (colon). We confirmed statistically significant differences between CD and UC in weight loss, weakness, mucosal changes in the oral cavity, and upper part of the digestive tract, as well as perianal abnormalities. The change in daily activity, fatigue, and decreased exercise tolerance was characteristic for more than 72% of patients with CD and only 26% of patients with UC. Weight loss was observed in over half of children with CD and less than 24% in children with UC (Figure 1 and Figure 2).

Our results are comparable to other publications. We could not detect any new trends, e.g., change of the frequency in the location or the course of CD [5,8,13]. Moreover, the pattern of symptoms (diarrhoea, abdominal pain, extraintestinal manifestations) does not seem to change in the population of IBD patients of Lower Silesia compared to previous studies. The abdominal pain in both analyses was most frequently seen in the group of patients with CD (100% previously vs. 82% in the present study), whereas blood in stool was observed in the group with UC (85% vs. 95.2%). The analysis also confirms that typical symptoms of IBD (diarrhoea, abdominal pain) cannot be used as discriminating features between CD and UC. Despite the fact that diarrhoea affected over 80% of children with UC (100% of the youngest) and only about 60% of patients with CD, the difference was not statistically significant. Abdominal pain reported more often in children with CD (82%) than in children with UC (76.2%) was also not statistically significant.

**Figure 6:**
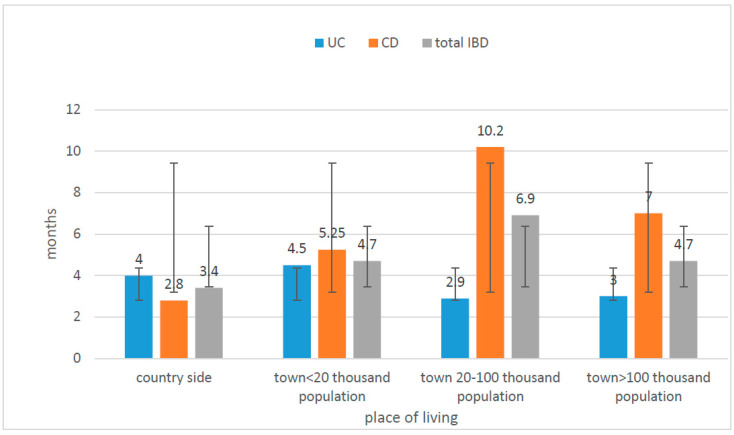
Correlation between time to diagnosis and place of residence: UC—ulcerative colitis; CD—Crohn’s disease; IBD—inflammatory bowel disease.

In our study, the period of time between first symptoms and diagnosis was much longer in the case of CD than UC (statistically significant, *p* = 0.008) and is probably connected with less specific symptoms presented by CD patients and a delay in reporting them to the doctors. We could observe this trend in all studied subgroups, taking into account sex and the place of living. There were no differences between children living in villages or small cities compared to large cities, and therefore, we do not believe the access to medical care could have an impact on the late diagnosis of CD. Moreover, when we analysed the whole group of IBD patients, not distinguishing them on the basis of CD and UC, those living in areas with lower population were diagnosed earlier, which suggests that they were sent to the gastroenterological department immediately after the first consultations at the level of basic medical care which significantly accelerated the process of reaching the final diagnosis (Figure 5 and Figure 6). We did not observe statistically significant differences between the frequency of UC incidence while taking into account the place of living. There were no statistically significant differences between villages/cities with less than 100,000 residents and cities up to 100,000 residents (52.3% and 56.4%, respectively). In conclusion, in order to answer the question of whether the incidence of IBD in the Lower Silesia region is more influenced by the individual lifestyle or environmental factors (e.g., pollution), it is necessary to conduct well-planned studies on a large group of patients.

Analysis of the relation between season of the year and onset of the symptoms revealed a little higher frequency of new cases in early winter and at the turn of spring and summer, respectively, 27.1% and 24.7%. However, the rest, almost 50% of patients, became ill equally over the summer and the fall; therefore, the seasonality of IBD in our region is still not clearly visible (Figure 3).

The analysis of the epidemiology of IBD in children from Lower Silesia was a retrospective work, it is the most serious limitation of the study. The collected and compared data concerned both patients diagnosed with the disease in our centre and children recognised in other hospitals. Therefore, it was not always possible to estimate all parameters due to the lack of data. It can also be assumed that not all patients diagnosed with IBD within the three analysed years were finally referred to our centre, and therefore, the epidemiological data may be incomplete. The small size of individual age groups, especially the youngest, limited the possibilities of statistical analysis.

However, these restrictions do not reduce the value of work. Determining the incidence of IBD seems to be infinitely important, especially in the paediatric group.

## 7. Conclusions

Our research confirms the upward trend in the incidence of IBD observed especially in developed countries. The increase in the incidence of Crohn’s disease, in particular, and the fact that the time from the first symptoms to the diagnosis is longer than in ulcerative colitis emphasises that it is often an insidious disease. The need to consider Crohn’s disease as a frequent and possible diagnosis in children with limited symptoms is crucial to protect against late recognition and severe complications. The incidence of inflammatory bowel diseases in the study population was gender independent, and the time of diagnosis was shorter in the village and small-town inhabitants, compared to urban inhabitants.

There is a constant need to research the IBD incidence trend in different age groups and geographies, including the impact of environmental factors and access to medical care, in order to better understand the etiological factors of IBD.

## Figures and Tables

**Table 1:** Age, gender, and time to diagnosis.

Diagnosis	Number of Children N	Age at the Time of Diagnosis (In Years)						Time from First Symptoms to Diagnosis (Months)		IBD in Family Number of Children
		From–To	Mean Age/SD	Girls		Boys				
				Number of Children	Mean Age/SD	Number of Children	Mean Age/SD	From–To	Mean Lenght/SD	
UC	42 (51.9%)	3.6–17.7	13.2/3.6	25 (59.5%)	13.6/3.2	17 (38.4%)	12.2/4.2	0.25–17	3.7/3.2	3 (7.1%)
CD	39 (48.1%)	2.6–17.0	13.1/3.9	15 (40.5%)	13.9/2.7	24 (61.6%)	12.5/4.6	1–60	6.9/9.5	2 (5.1%)
IBD	81 (100.0%)	2.6–17.7	13.1/3.8	40 (49.4%)	13.8/2.8	41 (50.6%)	12.4/4.4	0.25–60	5.8/8.0	5 (6.2%)

**Table 2:** Location, nutritional statement, endoscopic, and clinic activity at the time of diagnosis of UC.

	Extent of Changes	Number of Patients (Total 42)
Paris classification (UC)	E1: ulcerative proctitis	11
	E2: Left-sided UC (distal to splenic flexure)	18
	E3: Extensive (hepatic flexure distally)	4
	E4: Pancolitis (proximal to hepatic flexure)	9
Mayo score (UC)	Endoscopic severity of changes	
	Mayo 1: erythema, decreased vascular pattern, mild friability	7
	Mayo 2: marked erythema, absent vascular pattern, friability, erosions	28
	Mayo 3: spontaneous bleeding, ulceration	7
Paediatric ulcerative colitis activity index (PUCAI)	Clinic activity (points)	
	<10	5
	10–34	24
	35–64	8
	65–85	5
Nutritional statement	Body mass index BMI (kg/m^2^)	
	<15	2
	15–17.9	16
	18–20.9	14
	21–25	7
	>25	3

**Table 3:** Location, clinic, and endoscopic activity of changes at the time of diagnosis of CD.

Paris Classification (CD)	Location of Changes	Number of Patients (Total 39)
	L1: terminal ileal/limited caecal diseases	9
	L2: colonic	15
	L3: ileocolonic	13
	L4: isolated upper disease	2
SES-CD	Endoscopic severity of changes (points)	
	<5	11
	5–10	8
	>10	16
	Evaluated outside the clinic—lack of date	4
PCDAI	Clinic activity (points)	
	<10	8
	10–25	18
	26–50	10
	>50	3
Nutritional statement	Body mass index BMI (kg/m^2^)	
	<15	4
	15–17.9	20
	18–20.9	12
	21–25	2
	>25	1

**Table 4:** Incidence of IBD in the paediatric population, Lower Silesia, in 2016–2018.

Diagnosis	UC	CD	IBD		IBD	
					Boys	
					Girls	
Number of new cases in 2016–2018	42	39	81		41	
					40	
Number of children aged 0–19 (years) in the region of Lower Silesia	544,201					
	Boys (M)			277,941		
	Girls (F)			266,260		
Incidence rate—the number of IBD children aged 0–19 years, per 100 000, per year	2.57	2.38	4.96		4.9	5.0

**Table 5:** Frequency of symptoms in children with newly diagnosed IBD.

Symptom	UC *n* (%)	CD *n* (%)	*p*-Value
Diarrhoea	34 (89.95)	24 (61.54)	0.0528
Abdominal pain	32 (76.15)	32 (82.05)	0.5175
Blood in stool	40 (95.24)	2 (4.76)	0.00002
Weakness	11 (26.19)	28 (71.79)	0.00004
Weight loos	10 (23.81)	20 (51.28)	0.0105
Joints involvement	7 (16.67)	9 (23.08)	0.469
Skin changes	0 (0)	5 (12.82)	0.0532 *
Mucosal changes in oral cavity	1 (2.38)	11 (28.21)	0.0011
Perianal changes	0 (0)	11 (28.21)	0.00021

UC—ulcerative colitis; CD—Crohn’s disease; * *p*-value chi-square with Yates correction.

**Table 6:** Correlation between type of IBD and place of residence.

Place of Residence		Number of Patients				Statistical Analysis
		CD (*n* = 39)	%	UC (*n* = 42)	%	Test χ^2^
village		9	23.08	7	16.67	χ^2^ = 3.139
	town < 20,000 residence	8	20.51	15	35.71	Df = 3
	city 20—100,000 residence	15	38.46	11	26.19	*P* = 0.3707
	city >100,000 residence	7	17.95	9	21.43	

CD—Crohn’s disease; UC—ulcerative colitis.

## Data Availability

Data available in a publicly accessible repository that does not issue DOIs.
